# Rimming Plates

**Published:** 1858-04

**Authors:** S. Wardell


MR. JNO. T. TOLAND—DEAR SIR:—I wish to make a few remarks to your friends, through the medium of your "Reporter," on the subject of riming upper plates. My process is as follows:

After I have the teeth ground in and arranged just right, I place my plate with teeth on it on a single plaster cast, then mix some plaster about the stiffness of mortar, and place it with a spatula around the cast, and half way upon the teeth, so as to have it perfectly adapted around the teeth.

When the plaster is *hard*, I remove the teeth one at a time, and add more plaster, on top of the first and around the back part of the plate, being careful not to encroach too much, but barely touch the plate, this wall must rise about an inch above the plate. Mix a small quantity of whiting with water, about the thickness of cream, and with a camels hair pencil, paint the plate with the mixture to prevent the metal from adhering to it, place the cast with the plate, and "wall" in some places where it will become dry, pour in your metal on the plate, and within the wall.

I use what is called "Dorset's Metal" or "fusible alloy," *which* is composed of,

| | | |
|---|---|---|
| Bismuth, | 8 | parts, |
| Tin, | 4 | " |
| Lead, | 5 | " |

It fuses at a very low heat, and flows smooth and sharp when cold. Break away the wall, and if perfect, the metal cast will be a fac simile of the buts of your teeth as they were arranged on the plate. The strip which I use for a rim, is cut from my plate in a *circular form*, and is much more easily adapted than a straight strip could be. With this strip I begin at the *center* of the plate and strip, and cement them together, then imbed one end of the plate, and strip in plaster, as soon as it becomes sufficiently dry, I solder it at the center, using my metal cast as a guide to place my rim on the plate, bend the rim around a little at a time and solder, holding the rim to the plate, by means of iron wire, clamps, or plaster, as is most convenient. I do *not* solder continuously as I proceed, but merely attach the rim to the plate in spots, until I am sure that I have everything right, then solder all along, being careful to use but little solder, to avoid filling up the space into which the teeth are to fit.

After soldering, I pickle my plate to free it from the borax, then place the plate on the riming die, and with a small hammer, strike the rim up close to the cast, if the case has become sprung, I have only to place it on the zinc cast, and the riming die on top of it, when a blow with the hammer brings it back to its place, when the teeth can be re-arranged in their proper places, and soldered to the plate.

The above process is attended with more certain success than any other method that I have ever tried. S. W.

N. B.—I think the "cheoplastic metal" would do well for the above process.

NOTE.—No doubt the cheoplastic metal would work admirably, as it runs smooth and sharp; but we are inclined to think the profession would find it rather *costive !*—[ED.

